# Is [F-18]-fluorodeoxy-D-glucose positron emission tomography of value in the management of patients with craniofacial bone sarcomas undergoing neo-adjuvant treatment?

**DOI:** 10.1186/1471-2407-14-23

**Published:** 2014-01-15

**Authors:** Anna Maria Frezza, Tim Beale, Jamshed Bomanji, Amrita Jay, Nicholas Kalavrezos, Palma Dileo, Jeremy Whelan, Sandra J Strauss

**Affiliations:** 1 The London Sarcoma Service, University College London Hospital, London, England; 2 Department of Medical Oncology, University Campus Bio-Medico, Rome, Italy; 3 UCL Cancer Institute, Paul O’Gorman Building, 72 Huntley Street, London WC1E 6BT, England

**Keywords:** ^18^FDG PET/CT, Neoadjuvant chemotherapy, Craniofacial bone sarcoma

## Abstract

**Background:**

We evaluated the role of ^18^FDG PET/CT used to assess response to preoperative chemotherapy in patients with primary craniofacial bone sarcomas.

**Methods:**

Fourteen patients with craniofacial bone sarcomas (13 osteosarcoma, 1 spindle cell sarcoma) were retrospectively evaluated. All patients received up to 6 cycles of preoperative chemotherapy followed by resection of the primary tumour. Response to treatment was assessed using MRI (RECIST criteria) and ^18^FDG PET/CT (EORTC guidelines), performed at least at baseline, after 2-4 cycles and pre-operatively.

**Results:**

The median baseline ^18^FDG PET/CT SUV was 10.2 (range 0-41); in 2 patients no uptake was detected. The preoperative ^18^FDG PET/CT, compared with the baseline, demonstrated a partial metabolic response in 7 patients (59%), complete metabolic response in 2 (16%) and stable metabolic disease in 3 (25%). In contrast, only two patients achieved a RECIST response on MRI: 10 (83%) had stable disease. One patient underwent early resection due to clinical progression after an initial response to treatment. This was confirmed by PET (SUV from 21 to 42) but not on MRI. Twelve of 14 patients (86%) had <90% histological necrosis in the resected tumour. At a median follow-up 23 months, 11 patients (79%) remain disease free, two had metastatic progression (14%) and 1 a local relapse (7%). The median DFS was 17 months. For those patients who achieved a response to preoperative ^18^FDG PET/CT the median DFS was 19 months (range: 1-66) compared with 3 months (range: 3-13) in those who did not (p = 0.01). In contrast, the median disease free survival (DFS) did not differ according to histological response (19 versus 17 months, >90% versus <90% necrosis, p = 0.45) or resection margins (19 months for R0 versus 18 months for R1, p = 0.2).

**Conclusion:**

^18^FDG PET/CT is more reliable than standard imaging in evaluating response to neo-adjuvant chemotherapy in craniofacial bone sarcomas, changed management in one patient, and in this small series, correlated better with patient outcome than histological response and resection margins. These results warrant prospective validation in a larger cohort of patients.

## Background

Primary craniofacial bone sarcomas (CBS) are a group of rare tumours, accounting for less than 10% of all bone sarcomas and less than 1% of primary head and neck malignancies. They are usually diagnosed in older population than that affected by extremity bone sarcoma, with a median age at the time of diagnosis of 30 to 40 years, and a male to female ratio of around 1.7:1 [1-3]. Conventional osteosarcoma is the most represented histological subtype, most commonly the chondroblastic variant, and the vast majority arise from the mandible and maxilla.

At present a consensus about the optimal management of CBS does not exist, although patients are usually managed with a multimodal approach. Standard therapy in patients with extremity osteosarcoma is 2 cycles of neoadjuvant chemotherapy followed by 4 further cycles given post–operatively. Due to the complexity and morbidity of surgery, patients with CBS treated within the London Sarcoma Service, in line with other centres, receive all 6 cycles of chemotherapy pre-operatively where possible [4-6]. Radiotherapy is given post-operatively if surgical margins are involved and a further resection is not possible [4-6]. Current available evidence suggests that negative surgical margins are the strongest predictor of survival for this disease and, unlike extremity osteosarcoma, the role of histological necrosis after preoperative chemotherapy is still to be determined [7].

In this context, the on-going radiological assessment of the response to neo-adjuvant chemotherapy is of paramount importance. To date, standard magnetic resonance imaging (MRI) scanning remains the gold standard modality used to determine the morphological features of the tumour, assess its extent to plan surgery and it is also routinely used to evaluate response to preoperative treatment according to RECIST criteria. However, there is evidence to suggest that it does not allow an accurate discrimination between responders and non-responders [8,9]. Previous studies evaluating the role of [F-18]-fluorodeoxy-D-glucose positron emission tomography (^18^FDG PET/CT) in predicting chemotherapy response in bone sarcoma of the extremity have shown promising results. In this setting, ^18^FDG PET/CT has been proven to be feasible, more reliable than MRI scan in the response assessment and more accurate than MRI in predicting histological necrosis [10].

The aim of this retrospective study was to determine the value of ^18^FDG PET/CT compared with standard MRI scan in assessing the response to preoperative chemotherapy and determining management in patients with primary CBS. We also aimed to assess a possible predictive role of ^18^FDG PET/CT response and its correlation with histological necrosis.

## Methods

### Patients

Fourteen consecutive patients with primary CBS treated at University College London Hospital, London Sarcoma Service, between January 2005 and December 2011, were included in this retrospective study. All patients received up to 6 cycles of standard preoperative chemotherapy followed by the resection of the primary tumour. The chemotherapy regimens used in the current study included MAP (methotrexate 12 g/m^2^, doxorubicin 75 mg/ m^2^, cisplatin 120 mg/m^2^) for fit patients under 40 years of age or AP regimen (doxorubicin 75 mg/ m^2^, cisplatin 100 mg/m^2^). This study was undertaken in accordance with the NHS Research Ethics Service Guidance and NHS Health Research Authority policies regarding use of anonymised patient data [11,12].

### Imaging assessment and evaluation of response

All patients had an MRI scan of the primary tumour and ^18^FDG PET/CT at diagnosis. Staging was completed with a dedicated CT of the chest and technetium bone scan. Response to treatment was assessed using both MRI scan and ^18^FDG PET/CT, performed after 2 to 4 cycles of treatment and pre-operatively.

The ^18^FDG PET/CT response was assessed according to EORTC guidelines [13]. Progressive metabolic disease (PMD) was defined by an increase in tumour standard uptake value (SUV) greater than 25% or the appearance of new uptake foci; stable metabolic disease (SMD) was be classified as an increase in tumour SUV of less than 25% or a decrease of less than 15%; partial metabolic response (PMR) was be classified as a reduction of a minimum of 15-25% in tumour SUV after one cycle of chemotherapy, and greater than 25% after more than one treatment cycle. Complete metabolic response (CMR) was defined by complete resolution of FDG uptake within the tumour volume.

The MRI response was evaluated according to RECIST 1.1 criteria [14]. The disappearance of all target lesions was considered a complete response (CR) while partial response (PR) was defined as a decrease in the sum of diameters of target lesions of at least a 30%, taking as reference the baseline sum diameters. Progressive disease (PD) was defined as the appearance of one or more new lesions or an increase in the sum of diameters of target lesions of at least a 20%, taking as reference the smallest sum on study; in addition to the relative increase of 20%, the sum must also demonstrate an absolute increase of at least 5 mm. Neither sufficient shrinkage to qualify for PR nor sufficient increase to qualify for PD was considered as stable disease (SD).

The resection specimen was examined by two independent pathologists. Response to the chemotherapy was assessed in terms of percentage of necrosis (greater or less than 90%). Status of resection margins was classified as negative (R0) and positive (R1) or close (<1 mm).

### Statistical analysis

Disease free survival (DFS) was calculated as the period from surgery to the first evidence of disease progression. Descriptive analysis was made using median values and range. Survival analysis was performed by Kaplan-Meier product-limit method and the differences in term of DFS according to ^18^FDG PET/CT response or pathological response were evaluated by the log-rank test. SPSS software (version 17.00, SPSS, Chicago, ILQ5) was used for statistical analysis. A *P* value of less than 0.05 was considered to indicate statistical significance.

## Results

### Patient population

Patient characteristics are summarised in Table 1. The median age was 40 years (range: 22 to 63), 9 (64%) were male and 5 (36%) female, with a male to female ratio of 1.8:1. Thirteen patients (93%) were affected by osteosarcoma, one (7%) osteoblastic, 10 (71%) chondroblastic and 2 (15%) fibroblastic, while 1 patient (7%) was affected by a high grade spindle cell sarcoma. Two patients (15%) presented with a radiotherapy induced osteosarcoma, with a median of 9 years (range: 6 to 12) interval from the previous radiotherapy treatment. The primary site was mandible in 9 (69%) patients and maxilla in 5 (31%). One patient had metastatic disease, with lung metastases only. Nine (69%) patients received MAP chemotherapy whilst AP was used in 5 (31%). The median number of preoperative chemotherapy cycles was 6 (range: 2-6).

**Table 1:** Patients characteristics

**Characteristic**	**Patients (%)**
Median age (years)	40 years (range: 22–63 )
Gender	
Male	9 (64%)
Female	5 (36%)
Histology	
Osteoblastic osteosarcoma	1 (7%)
Chondroblastic osteosarcoma	10 (71%)
Fibroblastic	2 (15%)
Others	1 (7%)
Radiation induced sarcoma	2 (14%)
Median time to previous radiotherapy (years)	9 years (range: 6–12)
Primary site	
Mandible	9 (64%)
Maxilla	5 (36%)
Preoperative chemotherapy regimen	
MAP	9 (64%)
AP	5 (36%)

MAP = methotrexate 12 g/m^2^, doxorubicin 75 mg/ m^2^, cisplatin 120 mg/m^2^.

AP = doxorubicin 75 mg/ m^2^, cisplatin 100 mg/m^2^.

### Assessment of response to treatment

**Figure 1:**
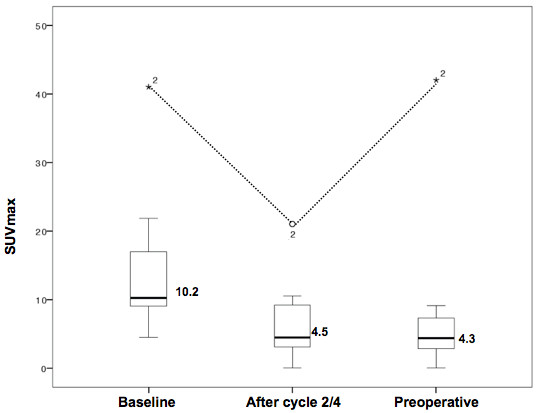
**Box and whiskers plot of median SUV max value at baseline, after 2–4 cycles of preoperative chemotherapy and preoperatively in 10 patients with positive PET scans at diagnosis.** *2 represents the only outlier value (SUV max 42, 21, 41).

The baseline ^18^FDG PET/CT scan demonstrated increased uptake in 12 of 14 (86%) patients. The median baseline SUV max value was 10.2 (range: 4.5-41). After 2-4 cycles of chemotherapy, the median SUV max value was 4.5 (range: 0-21). At the preoperative PET the median SUV max value was found to be 4.3 (range: 0-42) (Figure 1).

**Figure 2:**
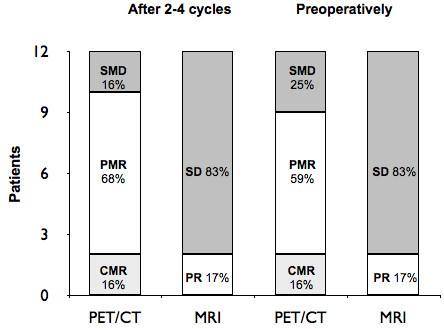
**Assessment of response to preoperative chemotherapy; determined by **^**18**^**FDG PET/CT and MRI scanning.** PET/CT response was assessed according to EORTC guidelines, SMD = 2, PMR = 8, CMR = 2 after 2–4 cycles; SMD = 3, PMR = 7, CMR = 2 preoperatively. MRI was assessed according to RECIST criteria, SD = 10, PR = 2 both after 2–4 cycles and preoperatively.

Among the 12 patients with positive uptake at diagnosis, 10 patients achieved a metabolic response to treatment after 2-4 cycles of chemotherapy; 8 (68%) achieved a PMR and 2 (16%) a CMR. Two patients (16%) had SMD and no PMD were recorded. The preoperative ^18^FDG PET/CT, compared with the baseline, demonstrated a PMR in 7 patients (59%), a CMR in 2 (16%) and SMD in 3 (25%). Two patients (17%) achieved a partial response on MRI according to RECIST criteria; 10 patients (83%) had SD (Figure 2). MRI however, demonstrated changes in signal characteristics with decreased enhancement and T2 signal in 5 patients (36%) suggestive of tumour response and an increased T2 signal due to necrosis in 2 patients (16%), including the one who achieved a partial response.

**Figure 3:**
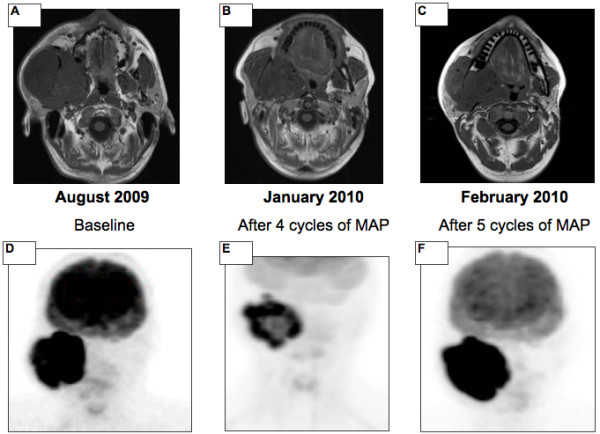
**The value of **^**18**^**FDG PET/CT value in determining the management of a patient with craniofacial bone sarcoma.** The initial response to treatment, after four cycles of MAP chemotherapy, is documented by both MRI (**A** versus **B**) and ^18^FDG PET/CT (**D** versus **E**). At the subsequent restaging, performed early due to worsening pain, the MRI scan **(C)** continued to demonstrate stable disease while the ^18^FDG PET/CT demonstrated overt disease progression with an increase in SUV from 21 to 42 **(F)**.

One patient initially had a partial response, demonstrated by both MRI scan and ^18^FDG PET/CT (SUV max from 42 to 21) after four cycles of MAP. After cycle number 5, due to increasing pain, a new ^18^FDG PET/CT was performed which demonstrated progressive metabolic disease (SUV max from 21 to 41). The MRI however remained stable according to RECIST criteria. Based on the ^18^FDG PET/CT result, the patient underwent early surgery (Figure 3). The baseline ^18^FDG PET/CT scan did not show any uptake in 2 patients (14%). Therefore, response to preoperative chemotherapy was evaluated through MRI scan, which remained stable in both patients throughout the treatment.

Pathological analysis demonstrated two patients had greater than 90% histological necrosis (14%). Both patients achieved a PMR to the PET while were stable at MRI. Resection margins were found to be clear in 12 patients (86%) and positive in 2 (14%). The first patient had a positive bone margin while in the second the buccal margin was involved. Both patients underwent a re-excision with clear margins.

### Outcome

With a median follow-up of 23 months, 11 patients (79%) remain disease free. One patient (7%) had metabolic progression of the primary tumour after 5 cycles of MAP, underwent early surgery (necrosis < 90%, clear margins) and experienced a local recurrence after 3 months. Two patients had metastatic progression (14%) with lung and lung and pancreatic metastases respectively at 3 and 15 months after completing therapy. None of the three patients who relapsed had positive margins at resection.

**Figure 4:**
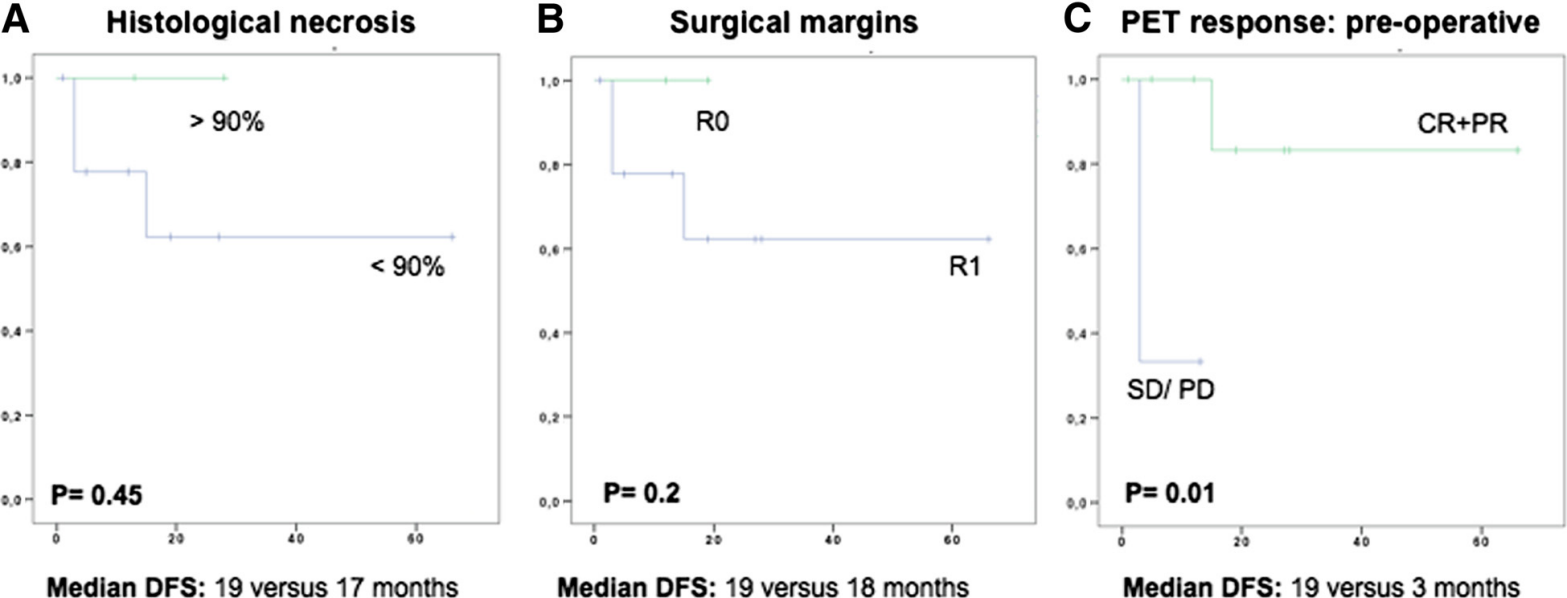
**Disease-free survival in patients with craniofacial bone sarcomas.** Kaplan Meier curves demonstrating disease-free survival in patients with craniofacial cone sarcomas according to histological necrosis **(A)**, status of resection margins **(B)** and preoperative ^18^FDG PET/CT **(C)** response.

Median disease free survival (DFS) was 17 months (range: 1-66). The median DFS in those patients who achieved an overall PET response (preoperative compared with baseline) was 19 months (range: 1-66) compared with 3 months in those who did not (range: 3-13; p = 0.01). Conversely, the median DFS did not differ according to histological response: 19 months for patients with a histological necrosis greater than 90% (range: 13-25) and 17 months (range: 1-66) for those with <90% (p = 0.45). Resection margins were also found not to correlate with survival (19 months for R0 versus 18 months for R1, p = 0.2) and none of the 2 patients with positive resection margins developed recurrent disease. Kaplan Meier survival analysis is demonstrated in Figure 4. The two patients with no baseline ^18^FDG PET/CT uptake at the primary site underwent surgery with clear margins and < 90% necrosis in both cases. DFS was 29 and 12 months respectively and both patients are alive and disease free.

## Discussion

This is the first report demonstrating the value of ^18^FDG PET/CT in assessing the response to preoperative chemotherapy in patients with craniofacial bone sarcomas. In this setting, due to the complexity and morbidity of surgery, all chemotherapy is given pre-operatively where possible and therefore ongoing assessment of response to therapy is important. In this small retrospective study, ^18^FDG PET/CT appears superior to the standard imaging modality, MRI in distinguishing responders from non responders. In 10 (86%) of 12 patients, MRI scans performed after 2-4 cycles and pre-operatively demonstrated stable disease according to RECIST criteria. A proportion of patients did have changes in signal characteristics that may be indicative of response, such as focal non-enhancing areas that demonstrate increased T2 signal in keeping with necrosis and decreased enhancement and T2 signal suggestive of fibrosis. However, these criteria are not standardised. In contrast, the preoperative ^18^FDG PET/CT identified a metabolic response in 9 patients (CMR in 7 patients, 16%, PMR in 2, 59%) and SMD in 3 patients (25%). The ability to quantify responses identified by the ^18^FDG PET/CT is extremely useful in guiding the clinician through the patient’s treatment and in selecting the optimal timing of resection. The impact of ^18^FDG PET/CT in the management of CBS patients is exemplified by the clinical case represented in Figure 4, where disease stability demonstrated by the MRI scan may have led to the continuation of chemotherapy while the striking increase in the SUV detected by the ^18^FDG PET/CT determined the decision for early surgery.

MRI plays an important role in the management of bone sarcoma. It is the optimal imaging modality to define the extent of the lesion within the bone as well as within the soft tissues, to detect skip metastases and to evaluate anatomic relationships with surrounding structures [15]. These features make MRI the gold standard in the initial staging of this disease, particularly when the tumour occurs in complex anatomic regions such as head and neck. Furthermore, providing excellent soft-tissue contrast, MRI is also essential for planning of surgical resection margins [16,17].

MRI has also been used routinely in the assessment of the response to preoperative chemotherapy, which is of paramount importance in the management of patients with CBS, who receive all the chemotherapy preoperatively where possible. However, the evaluation of changes in size according to RECIST criteria is not reliable in distinguishing response from non response; in a recent analysis of patients with bone sarcomas compared to histological response, MRI demonstrated a sensitivity of 81%, specificity of 68%, and an accuracy of 75% [8]. To overcome these limitations, the potential value of other imaging techniques, including diffusion-weighted (DW-MR) and dynamic contrast enhanced (DCE-MR) MRI and ^18^FDG PET/CT, that assess tumour cellularity and vascularity are being assessed [18,19].

^18^FDG PET/CT is a non-invasive functional imaging modality that can detect changes in tissue metabolism usually preceding structural ones and which may more accurately identify viable residual tumor [9,10]. Previous prospective studies conducted in extremity osteosarcoma clearly demonstrated the feasibility of ^18^FDG PET/CT in this setting and its superiority to standard MRI in predicting histological response to preoperative chemotherapy [10,20]. Furthermore, a large series reported by Hawkins et al. assessed the potential prognostic value of ^18^FDG PET/CT response [21]. That study, which prospectively evaluated 40 patients with extremity osteosarcoma, demonstrated that a low SUV value after completion of neoadjuvant chemotherapy strongly correlated with PFS (P = 0.021). In particular, the 4-year PFS was reported to be 73% for those patients whose preoperative SUV was < 2.5 compared with 39% for those with a SUV ≥ 2.5. Similar data have also been previously reported by the same group regarding the prognostic role of ^18^FDG PET/CT response to preoperative chemotherapy in Ewing sarcoma [22]. Despite these results, no data are available examining the use of ^18^FDG PET/CT in CBS, and the possible role of functional imaging in determining the management of these patients has been poorly investigated.

The survival analysis in this group of patients demonstrated an encouraging outcome, with a median DFS of 17 months and 79% of patients remain disease free after a median follow up of 23 months. This is in line with recent analyses of outcome in this group of patients treated with multimodality therapy [23]. Interestingly, although a trend to greater disease free survival was observed in patients with complete resections and greater than 90% histological necrosis after preoperative chemotherapy, in this small group of patients the difference was not found to be significant (p = 0.2 and p = 0.45 respectively). These results suggest that the predictive role of histological necrosis, which is well established for extremity bone sarcoma after 2 cycles of therapy, may not be of equal value for CBS [24]. Conversely, preoperative PET response predicted disease-free survival in CBS patients, with a median DFS of 19 months in those patients achieving a ^18^FDG PET/CT response compared with 3 month in those who did not (p = 0.01). The value of these results is limited by the small sample size and the retrospective nature of the study.

## Conclusions

This small retrospective study demonstrated that standard MRI is inadequate in assessing response to preoperative chemotherapy in CBS patients. Conversely, the use of ^18^FDG PET/CT is feasible, reliable and is able to distinguish responders from non responders. In addition, in this group of patients, ^18^FDG PET/CT response demonstrated a greater correlation with outcome than resection margins or histological response to treatment. Validation in a prospective study with a larger cohort of patients is warranted.

## Abbreviations

CBS: Craniofacial bone sarcomas; MRI: Magnetic resonance imaging; DW-MR: Diffusion-weighted magnetic resonance; DCE-MR: Dynamic contrast enhanced magnetic resonance; FDG PET/CT: [F-18]-fluorodeoxy-D-glucose positron emission tomography; SUV: Standard uptake value; CMR: Complete metabolic response; PMR: Partial metabolic response; SMD: Stable metabolic disease; PMD: Progressive metabolic disease; PR: Partial response; PR: Partial response; SD: Stable disease; DFS: Disease free survival.

## Competing interests

The authors declare that they have no competing interests.

## Authors’ contributions

JW, SS, AMF conceived the study and the design. AMF carried out data collection and statistical analysis. AMF, TB, AJ, JB, NK, SM, PD, JW and SS helped to draft the manuscript. All authors read and approved the final manuscript.

## Pre-publication history

The pre-publication history for this paper can be accessed here:

http://www.biomedcentral.com/1471-2407/14/23/prepub

## References

[B1] Ha PK Eisele DW Frassica FJ Zahurak ML McCarthy EF Osteosarcoma of the head and neck: a review of the Johns Hopkins experience Laryngoscope 1999 109 6 964 969 10.1097/00005537-199906000-00023 10369291

[B2] Laskar S Basu A Muckaden MA D’Cruz A Pai S Jambhekar N Tike P Shrivastava SK Osteosarcoma of the head and neck region: lessons learned from a single-institution experience of 50 patients Head Neck 2008 30 8 1020 1026 10.1002/hed.20820 18383528

[B3] Oda D Bavisotto LM Schmidt RA McNutt M Bruckner JD Conrad EU 3rd Weymuller EA Jr Head and neck osteosarcoma at the University of Washington Head Neck 1997 19 6 513 523 10.1002/(SICI)1097-0347(199709)19:6<513::AID-HED9>3.0.CO;2-1 9278760

[B4] Smeele LE Kostense PJ van der Waal I Snow GB Effect of chemotherapy on survival of craniofacial osteosarcoma: a systematic review of 201 patients J Clin Oncol 1997 15 1 363 367 8996163 10.1200/JCO.1997.15.1.363

[B5] Thiele OC Freier K Bacon C Egerer G Hofele CM Interdisciplinary combined treatment of craniofacial osteosarcoma with neoadjuvant and adjuvant chemotherapy and excision of the tumour: a retrospective study Br J Oral Maxillofac Surg 2008 46 7 533 536 10.1016/j.bjoms.2008.03.010 18436357

[B6] Guadagnolo BA Zagars GK Raymond AK Benjamin RS Sturgis EM Osteosarcoma of the jaw/craniofacial region: outcomes after multimodality treatment Cancer 2009 115 14 3262 3270 10.1002/cncr.24297 19382187

[B7] Patel SG Meyers P Huvos AG Wolden S Singh B Shaha AR Boyle JO Pfister D Shah JP Kraus DH Improved outcomes in patients with osteogenic sarcoma of the head and neck Cancer 2002 95 7 1495 1503 10.1002/cncr.10849 12237918

[B8] Miwa S Taki J Yamamoto N Shirai T Nishida H Hayashi K Tanzawa Y Kimura H Takeuchi A Igarashi K Ooi A Tsuchiya H A novel combined radiological method for evaluation of the response to chemotherapy for primary bone sarcoma J Surg Oncol 2012 106 3 273 279 10.1002/jso.23074 22389049

[B9] Denecke T Hundsdorfer P Misch D Steffen IG Schonberger S Furth C Plotkin M Ruf J Hautzel H Stover B Assessment of histological response of paediatric bone sarcomas using FDG PET in comparison to morphological volume measurement and standardized MRI parameters Eur J Nucl Med Mol Imaging 2010 37 10 1842 1853 10.1007/s00259-010-1484-3 20505933

[B10] Bajpai J Kumar R Sreenivas V Sharma MC Khan SA Rastogi S Malhotra A Gamnagatti S Safaya R Bakhshi S Prediction of chemotherapy response by PET-CT in osteosarcoma: correlation with histologic necrosis J Pediatr Hematol Oncol 2011 33 7 e271 e278 22193290 10.1097/MPH.0b013e31820ff78e

[B11] NHS National research ethics service guidance http://www.nres.nhs.uk/applications/guidance/

[B12] NHS health research authority http://www.hra-decisiontools.org.uk/research/index.html

[B13] Young H Baum R Cremerius U Herholz K Hoekstra O Lammertsma AA Pruim J Price P Measurement of clinical and subclinical tumour response using [18F]-fluorodeoxyglucose and positron emission tomography: review and 1999 EORTC recommendations. European Organization for Research and Treatment of Cancer (EORTC) PET Study Group Eur J Cancer 1999 35 13 1773 1782 10.1016/S0959-8049(99)00229-4 10673991

[B14] Eisenhauer EA Therasse P Bogaerts J Schwartz LH Sargent D Ford R Dancey J Arbuck S Gwyther S Mooney M New response evaluation criteria in solid tumours: revised RECIST guideline (version 1.1) Eur J Cancer 2009 45 2 228 247 10.1016/j.ejca.2008.10.026 19097774

[B15] Heck RK Jr Peabody TD Simon MA Staging of primary malignancies of bone CA Cancer J Clin 2006 56 6 366 375 10.3322/canjclin.56.6.366 17135693

[B16] Spina V Romagnoli R Manfrini M Cerofolini E Capanna R Gaiani L Calandra Buonaura P Picci P Campanacci M Magnetic resonance for the study of osteosarcoma Radiol Med 1991 81 1–2 29 37 2006331

[B17] Onikul E Fletcher BD Parham DM Chen G Accuracy of MR imaging for estimating intraosseous extent of osteosarcoma AJR Am J Roentgenol 1996 167 5 1211 1215 10.2214/ajr.167.5.8911182 8911182

[B18] Baunin C Schmidt G Baumstarck K Bouvier C Gentet JC Aschero A Ruocco A Bourlière B Gorincour G Desvignes C Colavolpe N Bollini G Auqier P Petit P Value of diffusion-weighted images in differentiating mid-course responders to chemotherapy for osteosarcoma compared to the histological response: preliminary results Skeletal Radiol 2012 41 9 1141 1149 10.1007/s00256-012-1360-2 22318350

[B19] Oka K Yakushiji T Sato H Hirai T Yamashita Y Mizuta H The value of diffusion-weighted imaging for monitoring the chemotherapeutic response of osteosarcoma: a comparison between average apparent diffusion coefficient and minimum apparent diffusion coefficient Skeletal Radiol 2010 39 2 141 146 10.1007/s00256-009-0830-7 19924412

[B20] Hawkins DS Rajendran JG Conrad EU 3rd Bruckner JD Eary JF Evaluation of chemotherapy response in pediatric bone sarcomas by [F-18]-fluorodeoxy-D-glucose positron emission tomography Cancer 2002 94 12 3277 3284 10.1002/cncr.10599 12115361

[B21] Hawkins DS Conrad EU 3rd Butrynski JE Schuetze SM Eary JF [F-18]-fluorodeoxy-D-glucose-positron emission tomography response is associated with outcome for extremity osteosarcoma in children and young adults Cancer 2009 115 15 3519 3525 10.1002/cncr.24421 19517457 PMC2716419

[B22] Hawkins DS Schuetze SM Butrynski JE Rajendran JG Vernon CB Conrad EU 3rd Eary JF [18 F]Fluorodeoxyglucose positron emission tomography predicts outcome for Ewing sarcoma family of tumors J Clin Oncol 2005 23 34 8828 8834 10.1200/JCO.2005.01.7079 16314643

[B23] Jasnau S Meyer U Potratz J Jundt G Kevric M Joos UK Jurgens H Bielack SS Craniofacial osteosarcoma experience of the cooperative German-Austrian-Swiss osteosarcoma study group Oral Oncol 2008 44 3 286 294 10.1016/j.oraloncology.2007.03.001 17467326

[B24] Bacci G Mercuri M Longhi A Ferrari S Bertoni F Versari M Picci P Grade of chemotherapy-induced necrosis as a predictor of local and systemic control in 881 patients with non-metastatic osteosarcoma of the extremities treated with neoadjuvant chemotherapy in a single institution Eur J Cancer 2005 41 14 2079 2085 10.1016/j.ejca.2005.03.036 16115755

